# Susceptibility Of Ph-Positive All To Tki Therapy Associated With Bcr-Abl Rearrangement Patterns: A Retrospective Analysis

**DOI:** 10.1371/journal.pone.0110431

**Published:** 2014-11-21

**Authors:** Yu Jing, Huiren Chen, Mingjuan Liu, Minhang Zhou, Yuelu Guo, Chunji Gao, Quanshun Wang, Honghua Li, Yu Zhao, Jian Bo, Wenrong Huang, Haiyan Zhu, Yongqing Zhang, Li Yu

**Affiliations:** 1 Department of Hematology and BMT center, Chinese PLA General Hospital, Beijing, China; 2 Department of Hematology, General Hospital of Beijing Military Region, Beijing, China; 3 Department of Hematology, the 309th Hospital of Chinese People's Liberation Army, Beijing, China; Emory University, United States of America

## Abstract

***Background*:**

Tyrosine kinase inhibitors (TKIs) have demonstrated success in the treatment of acute lymphoblastic leukemia (ALL) in patients that express BCR-ABL rearrangements (Philadelphia chromosome [Ph]). The current study aimed to assess the efficacy of TKIs and prognostic factors in the treatment of adults with Ph+-ALL.

***Methods*:**

In this multicenter retrospective study, the relationship between Ph+-ALL and treatment outcomes among Chinese patients receiving TKI-containing induction/consolidation chemotherapy was examined. A total of 86 Ph+-ALL patients were included and followed for 3.85 (0.43–9.30) years. Overall survival (OS) and event-free survival (EFS) were analyzed.

***Results*:**

A total of 86 Ph+-ALL patients (40 females and 46 males; median age: 34.0 years) were enrolled, including those with BCR/ABL transcripts 190 (n = 52), 210 (n = 25), and 230 (n = 2); BCR/ABL isoform determination was not available for 7 patients. Mortality was influenced by variable BCR/ABL transcripts and TKI administration, and BCR/ABL transcripts, hematopoietic stem cell transplantation (HSCT), and TKI administration were associated with the occurrence of events. The OS rate in the TKI administration group during steady state was significantly higher compared with those patients who did not receive TKI administration (*P* = 0.008), the EFS rate in the TKI administration group during steady state was significantly higher compared with those patients who did not receive TKIs (*P* = 0.012), and also higher than those with TKI salvage administration (*P* = 0.004). BCR/ABL transcripts 210 showed preferable OS and EFS compared with BCR/ABL transcripts 190 and 230 (*P*<0.05 for each).

***Conclusions*:**

The susceptibility of Ph+-ALL to TKI associated with the patterns of BCR-ABL rearrangement is demonstrated for the first time, thus adding another risk-stratifying molecular prognostic tool for the management of patients with Ph+-ALL.

## Introduction

Philadelphia chromosome positive (Ph+) acute lymphoblastic leukemia (ALL) affects approximately 25% of adults with ALL, in particular those older than 40 years of age [1]. Ph is the most common chromosomal abnormality in patients with ALL and is characterized by the BCR/ABL fusion gene [2]. The incidence of Ph+-ALL is associated with increased age: approximately 12–30% of patients aged 18–35 years and about 40–45% of patients aged 36–50 years. Greater than 50% of patients with ALL who are older than 60 years of age are Ph+ [3]–[5].

The poor survival of Ph+-ALL patients treated with chemotherapy alone (10%) has been substantially improved through the use of allogeneic hematopoietic stem cell transplantation (HSCT) in first complete remission (CR1) [6]–[10]. In patients achieving complete remission (CR) before transplantation, 40%–60% of patients experienced long-term survival [11]. Recently, the combination of TKIs, BCR/ABL kinase specific inhibitors, with induction and post-remission chemotherapy has significantly improved the long-term survival of ALL patients in CR [6]–[9]. Cure rates in adults with ALL have been improved in association with Imatinib treatment in combination to conventional chemotherapy [12]. Dasatinib, a novel ABL tyrosine kinase inhibitor, has been used for the treatment of relapsed and refractory Ph+-ALL and has been associated with significantly higher CR rate and lower toxicity than induction chemotherapy [7]. TKIs in combination with chemotherapy may achieve a higher CR rate compared with chemotherapy alone. CR rates have been reported to be as high as 90%–100% with survival rates also increased significantly [2].

Treatment of refractory and recurrent Ph+ chronic myeloid leukemia (CML) with TKIs has also been successful, as evidenced by molecular CR and long-term survival [13],[14]. However, despite the positive contribution of TKIs in the treatment of Ph+-ALL, HSCT remains the only established curative therapy. Incorporating TKIs into induction chemotherapy did not increase toxicity, but substantially improved remission rates and facilitated increased HSCT in CR1 [15],[16].

Taken together, due to the aggressive nature of the disease despite available treatment options, the clinical management of patients with PH+-ALL continues to be challenging. Patients continue to demonstrate complications in the remission/induction phase including a high rate of infection. Conventional chemotherapy often offers short-term clinical remission, whereas relapse is common unless patients receive HSCT. In this multicenter retrospective study, the efficacy of TKIs in combination with chemotherapy in the treatment of Ph+-ALL adults was examined, including the effect of HSCT timing on survival. Specifically, the association between different types of BCR/ABL rearrangements and treatment outcomes among patients receiving TKI-containing induction/consolidation chemotherapy was evaluated. The current study aimed to assess the efficacy of TKI and prognostic factors in the treatment of adults with Ph+-ALL.

## Materials And Methods

### Patients

In this multicenter retrospective study, a total of 86 Ph+ ALL patients were included and median follow-up time for 3.85 (0.43–9.30) years (follow up was performed during scheduled hospital visits or via telephone). The participating hospitals are Chinese PLA General Hospital, General Hospital of Beijing Military Region, Beijing, and the 309th Hospital of Chinese People's Liberation Army. All patients were less than 60 years of age at the time of study initiation, and all patients underwent at least one chemotherapy treatment. All treatments and study protocols were approved by the Institutional Review Board of Chinese PLA General Hospital. Written parental consents were obtained from patients.

### Therapeutic Protocol

Of 86 patients, 3 received induction chemotherapy with low dose VP (vincristine 1.5 mg/m^2^.d and prednisone 1 mg/kg.d), and other patients received ALL–like chemotherapy, including the following protocols: DOLP (Daunorubicin + vincristine + prednisone + L-asparaginase), IOLP (Idarubicin + vincristine + prednisone + L-asparaginase), and VDLP (Vincristine + daunorubicin + prednisone + L-asparaginase). In several patients, chemotherapy was performed using a hyper-CVAD protocol (Cyclophosphamide + vincristine + epirubicin + dexamethasone), CMOAP protocol (Cyclophosphamide + mitoxantrone + prednisone + vincristine + cytarabine), or an MOAP protocol (Mitoxantrone + prednisone + vincristine + cytarabine). After remission was achieved, the further treatment to choose consolidation chemotherapy or HSCT was according to patient age, disease severity, donor availability, attitude of relatives and agreement to study participation, and economic status.

Treatment with TKIs (imatinib [400–600 mg/day] or dasatinib [140 mg/day]) was determined by the physician and duration of treatment was determined by patients' tolerance to therapy. Patients who were treated with TKI in the induction phase and consolidation phase of chemotherapy were classified as the *non-salvage therapy group* (n = 48) and patients who received TKI after recurrence were classified as the *salvage therapy group* (n = 17). An additional 21 patients were not treated with TKI. Remission was defined as a reduction of cancer cells in the bone marrow to less than 5% of total bone marrow cells.

### Rna Extraction And Complementary Dna (cdna) Synthesis

Mononuclear cells were separated from bone marrow samples using Ficoll-Hypaque gradient centrifugation. Total RNA was extracted using Trizol Reagent (Invitrogen, Carlsbad, CA, USA) according to the manufacturer's instructions. Reverse transcription was performed using random hexamer primers (final concentration 5 ng/µl; Promega, USA).

### Real-Time Q-Pcr

TaqMan-based real-time Q-PCR technology was used. PCR reactions and fluorescence measurements were performed with an ABI PRISM 7500 real-time PCR system (PE Applied Biosystems, Foster City, CA, USA). BCR-ABL primers and probes that amplified both b3a2 and b2a2 junctions were designed using Primer Express software version 2.0. Sequences were listed in **Table 1**.

**Table 1:** PCR primers and probes used.

BCR-ABL-E1A2-F	5'- CGCAAGACCGGGCAGAT- 3'
BCR-ABL-E1A2-R	5' - AACGAGCGGCTTCACTCAGA -3'
BCR-ABL-E1A2-P	5'-FAM-ACGATGGCGAGGGC - 3'
BCR-ABL-B2A2-F	5' - GCATTCCGCTGACCATCAAT - 3'
BCR-ABL-B2A2-R	5' - GTCCAGCGAGAAGGTTTTCCT - 3'
BCR-ABL-B2A2-P	5'- FAM-AAGCCCTTCAGCGGC - 3'
BCR-ABL-B3A2-F	5' - GCTGACCATCAATAAGGAAGATGA - 3'
BCR-ABL-B3A2-R	5' - GATGCTACTGGCCGCTGAAG- 3'
BCR-ABL-B3A2-P	5'- FAM-CTCTATGGGTTTCTGAATGT - 3'

### Statistical Analysis

Primary study endpoints were OS, defined as the time from diagnosis with ALL until patient death or last follow-up, and EFS, defined as the time from diagnosis with ALL until the time of cancer progression, death, or last follow-up.

Categorical variables were reported as total number (n) and percentages. Continuous variables were reported as median and inter-quartile range. Cox proportional hazards models were used to examine the impact of prognostic factors on mortality and recurrence. The cumulative event-free rates (both OS and EFS rates) were performed using Kaplan-Meier estimates. A *P*-value <0.05 in the univariable Cox proportional hazards models were forward selected into the multivariable analysis. The Cox proportional hazard assumptions were assessed using the correlation coefficients between the Schoenfeld residuals compared with OS and EFS rank, respectively (Table S1). A two-tailed *P*<0.05 indicated statistical significance. All statistical analyses were performed using SPSS 15.0 statistical software (SPSS Inc, Chicago, IL, USA).

## Results

### Patient Characteristics

A total of 86 PH+-ALL patients (40 females and 46 males) were enrolled from April 2007 to October 2013. Median age for all subjects was 34.0 years (inter-quartile range: 22.0–42.0 years). Enrolled subjects included BCR/ABL transcripts 190 (n = 52), BCR/ABL transcripts 210 (n = 25), BCR/ABL transcripts 230 (n = 2). BCR/ABL isoform determination was not available for 7 patients. Detailed clinical characteristics of the patients are summarized in **Table 2**.

**Table 2:** Summary of Patient Characteristics.

		N = 86
Age (years)		34.0 (22.0, 42.0)
Gender	Female	40 (46.5%)
	Male	46 (53.5%)
BCR/ABL transcripts	210	25 (31.6%)
	190	52 (65.8%)
	230	2 (2.5%)
WBC (10^9^/L)		33.6 (8.2, 90.0)
Hemoglobin (g/d)		86.0 (74.0, 112.0)
Platelet count (10^9^/L)		57.0 (32.5, 121.0)
Bone marrow cells (%)		89.2 (72.4, 94.0)
ECOG	0	6 (7.8%)
	1	39 (50.6%)
	2	26 (33.8%)
	3	6 (7.8%)
Other genetic abnormality		19/49 (38.8%)
Other chromosomal abnormality		16/48 (33.3%)
HSCT	No transplantation	24 (28.6%)
	Not obtain CR before transplantation	36 (42.9%)
	Obtain CR before transplantation	24 (28.6%)
TKI	No administration	21 (24.4%)
	Administration in steady state	48 (55.8%)
	Salvage administration	17 (19.8%)

Data are presented as count and percentage except for age, WBC, Hemoglobin, Platelet, and Bone marrow cells are presented as median and inter-quartile range.

Data missing rate: WBC: 8 (9.3%), Hb: 9 (10.5%), PLT: 10 (11.6%), BMC: 0: 15 (17.4%), BCR/ABL type: 7 (8.1%), ECOG0: 9 (10.5%), HSCT: 2 (2.3%), Other genetic abnormality: 37 (43.0%), Other chromosomal abnormality: 38 (44.2%).

No significant relationship between patient demographics or clinical characteristics and occurring mortality was observed (**Table 3**), except for BCR/ABL transcripts and TKI administration. The influences of BCR/ABL transcripts and TKI administration on mortality were statistically significant using univariate analyses and remained statistically significant after multivariate analyses (**Table 4**). Patients with BCR/ABL transcripts 230 were more likely to be associated with death (HR = 7.834, *P* = 0.013) compared with those with BCR/ABL transcripts 210. Patients who were administered TKIs during steady state were less associated with death (HR = 0.349, *P* = 0.006) compared with those who did not receive TKIs. Patients included in the salvage TKI administration group did not demonstrate any significant benefit in mortality occurrence (*P*>0.05) compared with those who did not receive TKIs.

**Table 3:** Univariable Analysis: Contributing Factors for Mortality and Event Status.

		HR (95% CI) of mortality	P-value	HR (95% CI) of event	P-value
Age (year)		1.002 (0.979, 1.024)	0.886	1.013 (0.991, 1.034)	0.246
Gender		1.433 (0.768, 2.674)	0.258	1.369 (0.763, 2.458)	0.292
WBC (109/L)		1.001 (0.999, 1.004)	0.255	1.001 (0.999, 1.003)	0.287
Hemoglobin (g/d)		0.995 (0.984, 1.006)	0.375	1.000 (0.990, 1.011)	0.987
Platelet count (109/L)		0.998 (0.994, 1.002)	0.373	0.999 (0.996, 1.002)	0.541
Bone marrow cells (%)		1.014 (0.994, 1.034)	0.175	1.009 (0.991, 1.027)	0.348
ECOG	0–1	Reference		Reference	
	2–3	0.826 (0.424, 1.610)	0.575	0.802 (0.421, 1.528)	0.503
BCR/ABL transcripts	210	Reference		Reference	
	190	2.346 (1.022, 5.384)	0.044*	2.270 (1.071, 4.811)	0.033*
	230	6.366 (1.299, 31.199)	0.022*	4.612 (0.979, 21.713)	0.053
Other genetic abnormality		1.265 (0.590, 2.711)	0.545	1.533 (0.738, 3.184)	0.252
Other chromosomal abnormality		0.999 (0.430, 2.317)	0.997	1.170 (0.524, 2.613)	0.702
HSCT	No HSCT	Reference		Reference	
	Not obtain CR before HSCT	0.618 (0.302, 1.265)	0.188	0.583 (0.297, 1.144)	0.117
	Obtain CR before HSCT	0.490 (0.220, 1.092)	0.081	0.461 (0.215, 0.986)	0.046*
TKI administration	None	Reference		Reference	
	Administration in steady state	0.383 (0.187, 0.787)	0.009*	0.420 (0.209, 0.842)	0.014*
	Salvage administration	0.782 (0.360, 1.697)	0.534	1.143 (0.558, 2.343)	0.714
Side effects due to chemotherapy		0.881 (0.462, 1.678)	0.699	0.981 (0.536, 1.795)	0.949
Side effects due to TKI		0.862 (0.307, 2.420)	0.778	0.669 (0.240, 1.865)	0.442
Complication of infection		1.021 (0.550, 1.892)	0.948	0.979 (0.548, 1.747)	0.942
Complication of hemorrhage		1.588 (0.619, 4.070)	0.336	1.301 (0.513, 3.294)	0.579

*P<0.05 indicates a significant influence on the occurrence of event.

**Table 4:** Multivariate Analyses: Contributing Factors for Mortality and Event Status.

		HR (95% CI) of mortality	P-value	HR (95% CI) of event	P-value
BCR/ABL transcripts	210	Reference		Reference	
	190	2.236 (0.954,5.238)	0.064	2.623 (1.182,5.817)	0.018*
	230	7.834 (1.531,40.082)	0.013*	7.065 (1.405,35.523)	0.018*
TKI	No administration	Reference		Reference	
	Administration in steady state	0.349 (0.164,0.744)	0.006*	0.426 (0.203,0.892)	0.024*
	Salvage administration	0.750 (0.312,1.802)	0.519	1.391 (0.608,3.183)	0.435

*p<0.05 indicates a significant influence on the occurrence of event.

Three variables were identified as being significantly associated with the occurrence of events including BCR/ABL transcripts, HSCT, and TKI administration (**Table 3**). Patients with BCR/ABL transcripts 190 were more likely to experience an event compared with BCR/ABL transcripts 210 as identified using univariable analyses (HR = 2.270, *P* = 0.033). Patients who were treated with TKIs during steady state were less likely to experience an event (HR = 0.420, *P* = 0.014) compared to those patients who did not receive TKIs. Significantly preferable prognosis (mortality) was observed in patients who obtained CR prior to HSCT (HR = 0.461, *P* = 0.046). However, multivariate analyses did not show statistical significance, and the result was excluded from the final multivariable model.

In the multivariate analyses (**Table 4**), patients with BCR/ABL transcripts 190 and 230 were more likely experience an event compared with patients with BCR/ABL transcripts 210 (HR  = 2.623 and 7.065, both *P* = 0.018). Patients who received TKIs during steady state were less likely to experience an event compared with those patients who were not treated with TKIs (HR = 0.426, *P* = 0.024). The final multivariate model did not include HSCT since the observed results did not remain statistically significant.

### Tki Administration And Prognosis

**Figure 1:**
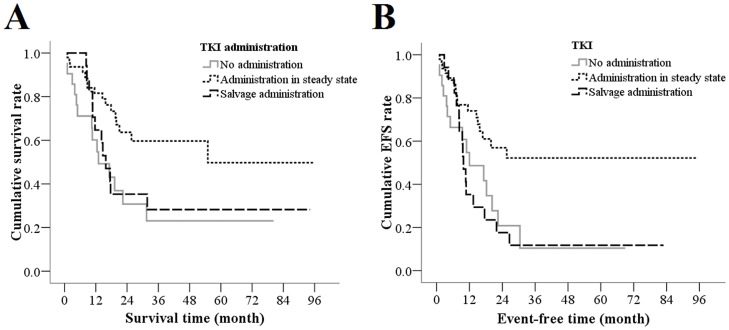
Prognosis for OS (A) and EFS (B) in Various TKI Administration Groups. The log-rank tests showed better prognosis in OS and EFS curves of those with TKI administration in steady stage compared to those without TKI administration (*P* = 0.008 and 0.012), and a significant difference was observed between the EFS curves of those with TKI administration in steady stage compared to salvage administration (*P* = 0.004).

The OS rate in TKI administration group during steady state was significantly higher compared with those patients who did not receive TKI administration (*P* = 0.008). No significant difference was observed in the survival curve of those with TKI salvage administration compared with those without TKI administration (**Figure 1A**). The 1-year survival rates for those patients who did not receive TKI administration, TKI administration during steady state, and for salvage administration were 60.2%, 81.6%, and 64.7%, respectively.

The EFS rate in the TKI administration group during steady state was significantly higher compared with those patients who did not receive TKIs (*P* = 0.012), and also higher than those with TKI salvage administration (*P* = 0.004; **Figure 1B**). No significant difference was observed between the EFS curves of those with TKI salvage administration and those without TKI administration. The 1-year EFS rates for those patients who did not receive TKI administration, TKI administration during steady state, and for salvage administration were 48.6%, 74.0%, and 35.3%, respectively.

### Bcr/abl Transcripts And Prognosis

**Figure 2:**
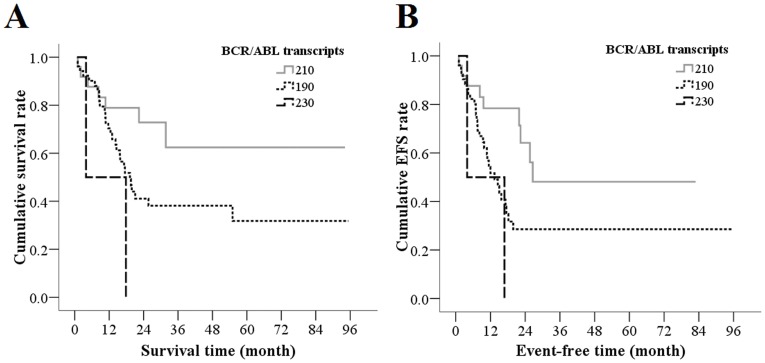
Prognosis for OS (A) and EFS (B) in Various BCR/ABL Transcripts Groups. The log-rank tests showed improved prognosis as demonstrated in the OS and EFS curves for those patients with BCR/ABL transcripts 210 compared to those with BCR/ABL transcripts 190 and 230 (*P* = 0.039 and 0.016 for OS curves, and 0.029 and 0.028 for EFS curves, respectively).

The Kaplan-Meier survival curves for those patients with BCR/ABL transcripts 210 showed improved OS compared with those patients with BCR/ABL transcripts 190 (*P* = 0.039) and 230 (*P* = 0.016; **Figure 2A**). The 1-year survival rates for those patients with BCR/ABL transcripts 210 and 190 were 78.9% and 70.4%, respectively. Only two subjects had BCR/ABL transcripts 230 –died at 3.9 and 17.8 months, respectively, after enrollment.

The Kaplan-Meier survival curves for those with BCR/ABL transcripts 210 showed improved EFS rates compared with those patients with BCR/ABL transcripts 190 (*P* = 0.029) and 230 (*P* = 0.028; **Figure 2B**). The 1-year EFS rates for those patients with BCR/ABL transcripts 210 and 190 were 78.4% and 51.7%, respectively. Only two subjects had BCR/ABL transcripts 230, and one died 3.9 months after enrollment, and the other recurred at 16.83 months after enrollment.

## Discussion

The use of TKIs for the treatment of Ph+-ALL has proven beneficial in both the induction and consolidation periods associated with chemotherapy. In order to assess the efficacy of TKIs and prognostic factors in the treatment of adults with Ph+-ALL, we conducted a multicenter retrospective study examining the relationship between Ph+-ALL and treatment outcomes among Chinese patients receiving TKI-containing induction/consolidation chemotherapy. A total of 86 Ph+-ALL patients were included and median follow-up time for 3.85 (0.43–9.30) years. In the present study, combined therapy (TKI plus chemotherapy) was evaluated in Ph+-ALL patients. TKI treatment varied relative to the timing of HSCT administration, thus, the influence of TKI treatment on OS and EFS, and its impact on the efficacy of HSCT therapy were examined. The main findings of the current study are BCR/ABL transcripts and TKI administration significantly influence mortality, and BCR/ABL transcripts, HSCT, and TKI administration are associated with the occurrence of events. In addition, the OS rate in the TKI administration group during steady state was significantly higher compared with those patients who did not receive TKI administration (*P* = 0.008), the EFS rate in the TKI administration group during steady state was also significantly higher compared with those patients who did not receive TKIs (*P* = 0.012), and also higher than those with TKI salvage administration (*P* = 0.004). BCR/ABL transcripts 210 showed improved OS and EFS compared with BCR/ABL transcripts 190 and 230 (*P*<0.05 for each).

In the present study, the main findings (OS and EFS after TKI administration and HSCT therapy) are similar to those reported in previous studies. The combined use of TKIs in the remission/induction phase has been shown to increase the 3-year OS to 72% in Ph+-ALL patients, but the 3-year OS was only 14% in patients without HSCT [17]. In the UKALL12/E2993 study, 3-year OS was 59% in patients with HSCT, and the prognosis of patients without HSCT was still poor despite TKI treatment [18].

There are also several novel conclusions resulting from the current study. Factors influencing the prognosis of these patients included: (1) the time of TKI treatment. Current results showed application of TKI at the first therapy and after first complete remission was associated with increased efficacy. (2) allogeneic hematopoietic stem cell transplantation. (3) application of TKI at recurrence or in the absence of remission did not influence long-term survival for the current patient population, despite allogeneic hematopoietic stem cell transplantation. (4) the BCR/ABL subtype is an important factor influencing the prognosis in patients with Ph+-ALL.

For patients undergoing HSCT, approximately 60% are tolerant to the early TKI treatment, while dose reduction or even discontinuation should be considered for some patients [19]. Additional studies are needed to clarify the role of early treatment with TKI, an option that may increase OS after HSCT. Worth noting, patients are tolerant to TKI at a reduced dose within 1 year after HSCT, which may also achieve improved OS [19]. Furthermore, there has been evidence supportive of TKI treatment after HSCT associated with the reduction in the incidence of disease recurrence and improved disease-free survival [7].

Several recent reports support the pre-emptive (prophylactic) use of TKIs after HSCT. More effective TKIs appear to increase molecular response rates, including dasatinib and imatinib.

After bone marrow transplantation, dasatinib administration for the purpose of prophylaxis against relapse has been associated with remission, dasatinib discontinuation resulted in molecular remission for 7 months, suggesting dasatinib may eradicate the minimal residual disease and prevent recurrence [20]. Following HSCT, the pre-emptive use of imatinib appears to reduce the relapse rate. Front-line imatinib-based therapy has increased the probability of patients undergoing HSCT by lowering the relapse rate [21]–[25]. Relapse before HSCT has been demonstrated to be significantly less frequent in patients pre-emptively treated with imatinib (3.5% versus 42.3%, *P* = 0.002), in addition, OS was superior to pre-emptive imatinib therapy [25].

After TKI treatment, whether the rapid reduction in BCR/ABL is predictive of a good prognosis remains controversial [26]. However, for patients receiving transplantation, detection of BCR/ABL before and after transplantation is still able to guide early treatment with the goal of preventing disease recurrence [27],[28]. The German multicenter study group for adult ALL (GMALL) evaluated the imatinib therapy in inducing BCR/ABL negativity and reducing the relapse rate in 27 minimal residual disease patients following HSCT [27]. Briefly, results demonstrated that BCR/ABL transcripts were undetectable in 52% of patients for a median of 1.5 months. All dasatinib-treated patients remained in remission, whereas three patients relapsed with discontinuation of imatinib. Taken together, failure to induce BCR/ABL negativity shortly after initiating imatinib was predictive of relapse (occurring in 92% of patients).

Different CML isoforms have distinct effects on patient prognosis, however, this phenomenon is not clear in ALL. Currently, the clinical significance of understanding different BCR/ABL isoforms is to clarify their roles in Ph+-ALL patients in the absence of TKI treatment and to compare patients with and without TKI treatment. Both P190 and P210 fusion proteins have been associated with increased tyrosine kinase activity, promotion of cell proliferation, and inhibition of cell apoptosis. In transgenic mice, results have shown the carcinogenic properties of the P190 fusion protein were more potent than that of the P210 fusion protein. However, the difference between the P210 fusion protein and P190 fusion protein is seldom reported in patients with leukemia. In 1993, Secker-Walker and Craig reported M-bcr patients were older with a lower white blood cell count compared with m-bcr patients, but there was no marked difference in the prognosis between groups [29]. Cimino et al. (2006) reported in a clinical trial that BCR/ABL transcript 190 was closely associated with a white blood cells count of <16×109/L and high CD33 expression at initial diagnosis, and there was no significant difference in the remission rate between study groups [30].

Different isoforms have been demonstrated to have an association with PH+-ALL. The IK6 isoform is correlated with blast cells, cytogenetic abnormalities, BCR-ABL1 transcripts, increased risk of relapse, shorter relapse-free survival and overall survival at diagnosis [31]. The PAX5 gene, although frequently rearranged in BCR-ABL1 PH+-ALL, which is not associated with outcome measures [32]. The p190 isoform has been demonstrated to be the only prognostic factor to positively impact the 5-year overall survival and disease-free survival rates in patients with PH+-ALL [30].

The efficacious role of TKI treatment in combination with chemotherapy has been confirmed in previous studies, however, complications remain, including those in the remission and induction phases of therapy, and a high incidence of infection. TKI in combination with different chemotherapies may cause non-hematologic toxicities including constipation, nausea, vomiting, peripheral neuropathy, liver dysfunction and sepsis [7],[33]. Thus, it is necessary to investigate low-dose induction chemotherapy in combination with TKI treatment which may achieve high CR rate, reduce the drug related side effects, and elevate the tolerance of patients to HSCT. Chemotherapy at a routine dose alone or in combination with TKI therapy may be associated with a high incidence of severe side effects. TKI therapy in combination with low dose induction chemotherapy may achieve high CR rate and high safety, but more studies with large sample size are required to confirm these findings. In addition, although the incidence of BCR/ABL transcript 230 was low, these patients had a poor prognosis, thus, studies with a larger sample size are required to investigate the prognosis of these patients. The current study reports findings from a Chinese population. To the best of our knowledge, similar studies in a more diverse population are not available. Future retrospective analyses should be carried out across populations.

Clinically, the current study demonstrated the optimal timing for TKI application is during the induction and remission phases of therapy. In induction phase, chemotherapy in combination with TKI therapy should be performed as soon as possible in Ph+-ALL patients, which may achieve high CR rate. Even in those patients who developed refractory recurrent ALL, TKI treatment is still able to achieve a favorable CR rate. Patients receiving HSCT have a higher rate of survival compared with those patients who did not receive HSCT. This observation is independent of chemotherapy alone or in combination with TKIs. However, the long-term survival is improved in patients who received chemotherapy and TKI in combination. The timing of TKI treatment had no influence on the therapeutic efficacy of HSCT. Transplantation after non-salvage therapy with TKI may significantly prolong OS, and in salvage therapy survival time after transplantation is similar to that in patients who did not receive HSCT. Patients achieving CR1 before transplantation have significantly reduced risk and have a better survival when compared with patients without CR1 before transplantation. Thus, the presence of CR1 before transplantation may significantly increase the survival rate after transplantation and patients without CR1 before transplantation may have a high mortality due to recurrence after transplantation.

TKI resistance is an important issue in Ph+-diseases, and the mechanisms underlying resistance in patients with Ph+-ALL are multifactorial. This study, for the first time, demonstrates susceptibility of Ph+-ALL to TKI associated with the patterns of BCR-ABL rearrangement, thus adding another risk-stratifying molecular prognostic tool for the management of patients with Ph+-ALL.

## Supporting Information

Table S1 **The correlation coefficients between the Schoenfeld residuals versus the ranks of survival time and event-free time.**
(DOC)
