# Supplementary material for: Susceptibility Of Ph-Positive All To Tki Therapy Associated With Bcr-Abl Rearrangement Patterns: A Retrospective Analysis
Source: PLoS One. 2014 Nov 21;9(11):e110431. doi: 10.1371/journal.pone.0110431 (PMC4240579; doi:10.1371/journal.pone.0110431)
Supplement: Table S1 — (DOC) [file pone.0110431.s001.doc]

**Supplementary table 1.** The correlation coefficients between the Schoenfeld residuals versus the ranks of survival time and event-free time.

|  | **Rank of survival time** | |  | **Rank of event-free time** | |
| --- | --- | --- | --- | --- | --- |
|  | **Pearson Correlation** | **P-value** |  | **Pearson Correlation** | **P-value** |
| **The Schoenfeld residuals** |  |  |  |  |  |
| Age | 0.041 | 0.796 |  | 0.234 | 0.113 |
| Gender | 0.007 | 0.967 |  | -0.200 | 0.179 |
| WBC | -0.248 | 0.127 |  | -0.230 | 0.143 |
| Hemoglobin | -0.095 | 0.564 |  | 0.023 | 0.884 |
| Platelet | -0.317 | 0.052 |  | -0.199 | 0.213 |
| Bone marrow cells | -0.200 | 0.256 |  | -0.085 | 0.621 |
| BCR/ABL transcripts dummy variable 1 | 0.136 | 0.422 |  | -0.059 | 0.713 |
| BCR/ABL transcripts dummy variable 2 | 0.075 | 0.660 |  | 0.082 | 0.610 |
| Other genetic abnormality | -0.018 | 0.928 |  | -0.124 | 0.523 |
| Other chromosomal abnormality | -0.110 | 0.601 |  | 0.051 | 0.799 |
| ECOG | -0.168 | 0.319 |  | -0.171 | 0.291 |
| HSCT dummy variable 1 | 0.079 | 0.626 |  | -0.051 | 0.738 |
| HSCT dummy variable 2 | 0.226 | 0.160 |  | 0.109 | 0.478 |
| TKI dummy variable 1 | 0.005 | 0.975 |  | -0.145 | 0.331 |
| TKI dummy variable 2 | 0.119 | 0.452 |  | 0.181 | 0.224 |
| Side effects due to chemotherapy | -0.068 | 0.670 |  | -0.052 | 0.730 |
| Side effects due to TKI | 0.026 | 0.870 |  | -0.041 | 0.787 |
| Infection | -0.172 | 0.276 |  | -0.139 | 0.352 |
| Hemorrhage | -0.254 | 0.104 |  | -0.173 | 0.244 |
